# Behaviorally-relevant features of observed actions dominate cortical representational geometry in natural vision

**DOI:** 10.21203/rs.3.rs-5478816/v1

**Published:** 2024-12-03

**Authors:** Jane Han, Vassiki Chauhan, Rebecca Philip, Morgan K. Taylor, Heejung Jung, Yaroslav O. Halchenko, M. Ida Gobbini, James V. Haxby, Samuel A. Nastase

**Affiliations:** 1 Department of Psychological and Brain Sciences, Dartmouth College, Hanover, NH, USA; 2 Department of Medical and Surgical Sciences (DIMEC), University of Bologna, Bologna, Italy; 3 Princeton Neuroscience Institute and Department of Psychology, Princeton University, Princeton, NJ, USA

## Abstract

We effortlessly extract behaviorally relevant information from dynamic visual input in order to understand the actions of others. In the current study, we develop and test a number of models to better understand the neural representational geometries supporting action understanding. Using fMRI, we measured brain activity as participants viewed a diverse set of 90 different video clips depicting social and nonsocial actions in real-world contexts. We developed five behavioral models using arrangement tasks: two models reflecting behavioral judgments of the purpose (transitivity) and the social content (sociality) of the actions depicted in the video stimuli; and three models reflecting behavioral judgments of the visual content (people, objects, and scene) depicted in still frames of the stimuli. We evaluated how well these models predict neural representational geometry and tested them against semantic models based on verb and nonverb embeddings and visual models based on gaze and motion energy. Our results revealed that behavioral judgments of similarity better reflect neural representational geometry than semantic or visual models throughout much of cortex. The sociality and transitivity models in particular captured a large portion of unique variance throughout the action observation network, extending into regions not typically associated with action perception, like ventral temporal cortex. Overall, our findings expand the action observation network and indicate that the social content and purpose of observed actions are predominant in cortical representation.

## Introduction

How do we understand the actions of others? From a computational standpoint, an observer must extract behaviorally relevant information—such as the goal of an action or its social significance—from dynamic visual patterns of bodily movements interacting with other agents or objects (Marr & Vaina, 1982; Vaina, 1983). Similar to other domains of vision (e.g., Rolls & Tovee, 1995; Haxby et al., 2001; Hung et al., 2005; Freiwald & Tsao, 2010) and action execution (e.g., Georgopoulos et al., 1986; Churchland et al., 2012), visual action understanding likely relies on a hierarchy of population codes, where information is encoded in the geometric relationships among distributed patterns of neural activity (Edelman, 1998; Kriegeskorte, Mur, & Bandettini, 2008; Haxby et al., 2014). At each stage of the processing hierarchy, these representational spaces are reshaped so as to disentangle higher-level features and more explicitly represent behaviorally relevant information (DiCarlo & Cox, 2007; DiCarlo et al., 2012; Kriegeskorte & Kievit, 2013). The similarity among these neural representations is thought to inform our perception of similarity and ultimately guide behavior (Nosofsky, 1984; Shepard, 1987; Ashby & Perrin, 1988; Edelman et al., 1998; Carlson et al., 2014; Ritchie et al., 2015; Cohen et al., 2017). In the current work, we investigated the representational geometries supporting human action understanding. To robustly estimate the geometric structure of these cortical representational spaces, we sample the space of naturalistic observed actions as comprehensively as possible.

A large body of work in both nonhuman primates (e.g., di Pellegrino et al., 1992; Gallese et al., 1996; Fogassi et al., 2005) and humans (e.g., Decety et al., 1999; Grafton & Hamilton, 2007; Caspers et al., 2010; Grosbras et al., 2012; Oosterhof et al., 2013; Urgesi et al., 2014) has charted out a network of cortical areas involved in action observation and understanding. This action observation network appears to unite several major cortical systems. A lateral visual pathway (Pitcher & Ungerleider, 2021) proceeding from early visual areas to lateral occipitotemporal (LO) cortex and superior temporal sulcus (STS) is thought to support action understanding and social perception. Subfields of the LO encode visual motion (Zeki et al., 1991; Tootell et al., 1995), tool use (Martin et al., 1996; Chao et al., 1999; Beauchamp et al., 2002), faces (Kanwisher et al. 1997; Haxby et al. 2000), body parts (Downing et al., 2001; Orlov et al., 2010), and multi-body configurations (Walbrin & Koldewyn, 2019; Abassi & Papeo, 2020), suggesting a pivotal role for LO in action understanding (Kable et al., 2002; Kalénine et al., 2010; Lingnau & Downing, 2015; Wurm et al., 2016, 2017; Wurm & Caramazza, 2022). The posterior superior temporal sulcus (pSTS) in the lateral pathway is implicated in the perception of biological motion (Grossman et al., 2000; Puce & Perrett, 2003; Russ & Leopold, 2015) and social interaction (Isik et al., 2017; Walbrin et al., 2018), and may interface with broader systems for social cognition (Deen et al., 2015). The lateral pathway is complemented by a parieto-frontal system comprising anterior intraparietal sulcus (aIPS) and ventral premotor (vPM) cortex (Rizzolatti & Sinigaglia, 2010). The aIPS is situated near the end of the dorsal “vision for action” pathway (Ungerleider & Mishkin, 1982; Ungerleider & Haxby, 1994; Milner & Goodale, 1995) and is thought to encode action goals (Fogassi et al., 2005; Hamilton & Grafton, 2006; Bonini et al., 2010; Oosterhof et al., 2010). This parietal system is closely intertwined with prefrontal areas, particularly vPM cortex, also associated with motor planning and execution (di Pellegrino et al., 1992; Gallese et al., 1996; Buccino et al., 2001; Nelissen et al., 2005; Oosterhof et al., 2012).

Much of this prior work is grounded in experimental contrasts between rudimentary actions—e.g., grasping an object to eat it or place it in a container (e.g., Fogassi et al., 2005)—and highly-controlled video stimuli, e.g., depicting only the grasping hand (e.g., Wurm et al., 2017). These paradigms do not fully capture the richness and complexity of real-world action understanding (Haxby et al., 2020; Nastase et al., 2020). Highly controlled experimental stimuli may artificially constrain neural responses (David et al., 2004) and can make it difficult to assess the relative contribution of different action features to neural activity in natural contexts (Haxby et al., 2020).

The use of dynamic, naturalistic action stimuli has revealed a surprisingly prominent role of ventral temporal (VT) cortex in the perception of dynamic, naturalistic action (Russ & Leopold, 2015; Nastase et al., 2017; Haxby et al., 2020; Russ et al., 2022). VT is located at the anterior part of the ventral object vision pathway (Ungerleider & Mishkin, 1982; Haxby et al., 1991, 1994; Ungerleider & Haxby, 1994) and has been historically associated with face and object processing in studies using static images (Kanwisher et al., 1997; Haxby et al., 2001; Kravitz et al., 2013; Grill-Spector & Weiner, 2014). More recent work has begun to address these limitations by exploring the neural representations of observed action and social interaction using dynamic and naturalistic stimulus paradigms (Huth et al., 2012; Russ & Leopold, 2015; Sliwa & Freiwald, 2017; Tarhan & Konkle, 2020; Lee Masson & Isik, 2021; Landsiedel et al., 2022; Shahdloo et al., 2022; McMahon et al., 2023).

The transition from highly-controlled experimental manipulations to naturalistic videos of real-world actions marks a significant step forward in the study of action representation. This paradigm shift, however, raises the question of how to quantify the structure of action features as they occur in real-world contexts. For example, recent work has tested bottom-up visual features (e.g., derived from deep neural networks) and human annotations of particular action features (e.g., “is the action directed at an object?”; Tarhan & Konkle, 2020; McMahon et al., 2023). To move toward a higher, more holistic level of action understanding, researchers have begun to adopt behavioral arrangement tasks (Goldstone, 1994; Kriegeskorte & Mur, 2012; Cichy et al., 2019), allowing participants to freely group action stimuli based on their perceived similarity (C. E. Watson & Buxbaum, 2014; de la Rosa et al., 2014; Tucciarelli et al., 2019; Dima et al., 2022, 2023). For example, recent work using behavioral arrangements of naturalistic action clips has highlighted the importance of social-affective features in organizing our understanding of real-world actions (Dima et al., 2022). This approach provides a relatively direct window onto the psychological “space” in which actions are organized (Shepard, 1987; Gärdenfors & Warglien, 2012), and promises to reveal the most cognitively relevant information underlying the representations of others’ behavior (Thornton & Tamir, 2021).

In the current study, we developed a condition-rich, naturalistic paradigm to investigate the neural representations supporting action understanding across a broad range of social and nonsocial actions. We presented participants with 90 video clips depicting real-world actions from 18 social and nonsocial action categories. This paradigm was designed to sample action space broadly, in hopes of yielding richly structured neural representational geometries across a variety of cortical areas. To quantify the neural representational geometries supporting action understanding, we developed nine different representational models, ranging from low-level visual models to verbal annotations to behavioral judgements of the similarity of the visual content of the videos and the purposes of the actions. We focus in particular on representational models derived from behavioral arrangement tasks capturing high-level judgments of the transitive and social goals of actions. We hypothesized (1) that these higher-level, more behaviorally relevant representational geometries would emerge at later stages of the cortical processing hierarchy, and (2) that behavioral judgments of the purpose of actions—their transitive goals and sociality—would generally outperform other models, including behavioral judgments of the visual content of the stimuli, as conveyed by still images. Briefly, we find that these more holistic behavioral judgments capture dramatically more variance in neural representational geometry than lower-level models, and extend beyond the canonical action observation network into areas like ventral temporal cortex and precuneus.

## Results

**Figure 1.**
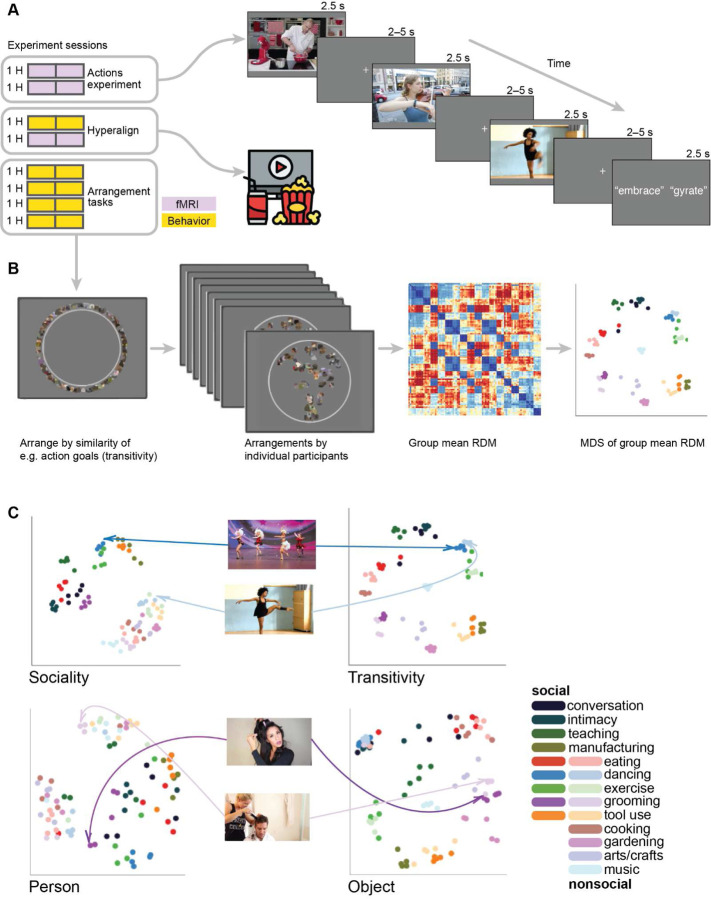
Experimental design for fMRI and behavioral data acquisition. (**A**) fMRI participants (*N* = 23) viewed 90 video clips of dynamic, naturalistic actions spanning 18 social and nonsocial action categories in a condition-rich design. Participants were intermittently presented with two action words and were asked to indicate by button-press which of the two words more accurately described the action depicted in the previous video (“gyrate” in the depicted example). In addition to the two action-viewing fMRI sessions, participants completed a third fMRI session where they viewed a ~1-hour naturalistic movie stimulus (the second half of *Raiders of the Lost Ark*). (**B**) A subset of participants (*N* = 17) completed five additional behavioral sessions where they performed five different multiple-arrangements tasks based on the action purpose (transitivity and sociality) of the video clips and visual content (people, objects, scenes) of still frames from the videos. Representational dissimilarity matrices (RDMs) were calculated based on the Euclidean distances in the two-dimensional arrangements and visualized using multidimensional scaling (MDS). (**C**) Participants produced different geometries based on the five different tasks. For example, a video depicting an individual dancer (light blue) was separated from a video depicting a group of dancers (dark blue) when participants arranged the stimuli based on sociality (top left); however, when participants arranged the stimuli based on transitivity, these two videos were clustered together (top right). When arranging static images according to the visual similarity of people, two stimuli depicting a man (light purple) and a woman (dark purple) with haircare devices were separated (bottom left); when arranging images according to the visual similarity of objects, these two stimuli were grouped together (bottom right).

**Figure 2.**
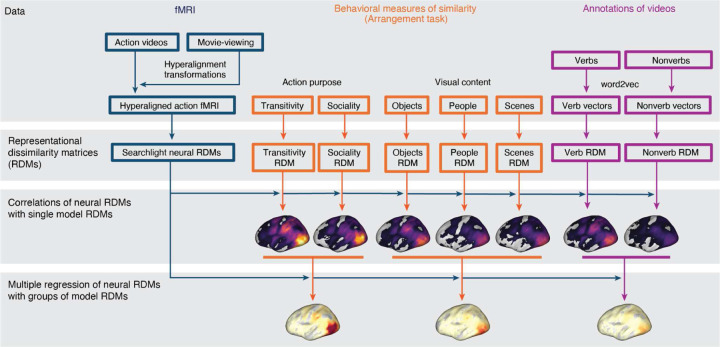
Schematic of pipeline for representational similarity analysis. fMRI data collected while participants viewed the action stimuli were hyperaligned based on responses to a separate 1-hour movie. Neural response patterns to the action stimulus were used to compute neural RDMs. A subset of participants performed behavioral arrangement tasks based on action purpose (transitivity, sociality) or visual content (objects, people, scenes); arrangements were converted into behavioral model RDMs. Annotations of videos with verb and nonverb labels were used to assign word2vec semantic embeddings to each action stimulus; verb and nonverb embeddings were used to construct semantic model RDMs. Spearman correlations were computed between different model RDMs and the neural RDMs. Multiple model RDMs were combined to jointly predict neural RDMs using multiple regression.

We used fMRI to measure brain activity in 23 participants while they viewed 90 different video clips depicting real-world actions in two scanning sessions (Fig. 1). Each clip was 2.5 seconds long and the 90 clips spanned 18 social and nonsocial action categories. The fMRI time series were submitted to a subject-level general linear model to estimate response patterns for each of the 90 stimuli, separately for the two different sessions. Using both a surface-based searchlight and targeted regions of interest (ROIs), we computed the Pearson correlation between local response patterns across the two scanning sessions to construct 90 × 90 split-data RDMs (Fig. 2). Prior to computing neural representational geometries, we used hyperalignment to better align cortical-functional topographies across individuals, based on data from a third session in which they viewed a naturalistic movie stimulus (the second half of *Raiders of the Lost Ark*). We used a whole-brain hybrid hyperalignment algorithm to transform response patterns from the action sessions in each individual into a common space based on both the response time series and functional connectivity during movie-viewing (Busch et al., 2021). These neural RDMs—the neural representational geometries supporting action perception across a variety of cortical regions—serve as the target for modeling in subsequent analyses.

### Models of representational geometry for action understanding

To understand the neural representational geometries supporting action understanding, we developed nine different representational models. These model RDMs serve as formal hypotheses about the structure of action representation to be evaluated against the neural RDMs (Kriegeskorte, Mur, & Bandettini, 2008; Kriegeskorte & Kievit, 2013). First, we constructed two low-level visual RDMs: (1) a *motion*-energy RDM capturing low-level dynamic visual features of video stimuli (Adelson & Bergen, 1985; A. B. Watson & Ahumada, 1985; Nishimoto et al., 2011); and (2) a *gaze* RDM constructed by computing the Euclidean distance between gaze trajectories acquired in a separate sample of participants (*N* = 17). Second, we constructed two semantic models based on an annotation of (3) *nonverbs* (nouns and adjectives) and (4) *verbs* depicted in the video stimuli (Huth et al., 2012). For each word, we obtained 300-dimensional word embeddings from the word2vec model (Mikolov et al., 2013). These embeddings geometrically capture the semantic relatedness of words based on their co-occurrence in large corpora of text. We averaged embeddings for the words assigned to each clip, then computed the pairwise cosine distance between average semantic vectors to construct nonverb and verb RDMs.

None of the aforementioned models directly assay human judgments of the relatedness of different actions. To address this, we constructed two additional groups of representational models based on behavioral judgments of similarity in a multiple-arrangements task (Goldstone, 1994; Kriegeskorte & Mur, 2012; Tucciarelli et al., 2019; Dima et al., 2022). Participants were instructed to arrange the stimuli according to perceived similarity along different criterial dimensions (Cichy et al., 2019). First, in three separate tasks, we presented participants with still images representative of each video and asked them to arrange the stimuli according to visual features having to do with the (5) *people*, (6) *objects*, or (7) *scene* depicted in the images. These tasks were selected to reflect three major features of cortical organization reported in the literature (Kanwisher, 2010). In these three tasks, participants were presented with still frames from the videos that could be clicked with the mouse to enlarge the image. Second, in two separate tasks, we presented participants with the video clips and asked them to arrange the stimuli according to two types of action content depicted in the videos: (8) *sociality*, capturing the nature of social interactions; and (9) *transitivity*, capturing the object- or goal-directed nature of the actions; (Wurm et al., 2017). In these two tasks, participants were presented with the same still frames, but the thumbnails could be clicked to enlarge and play the video clip. In the first trial of each task, all 90 stimuli were presented around the edge of a circle and participants were asked to move the stimuli into the circle and arrange them such that more similar videos were located nearer to each other, according to the task instructions. In 12 subsequent trials, pseudo-random subsets of 30 stimuli were presented and arranged.

To visualize the structure of these behavioral judgments, we use multidimensional scaling (MDS) (Torgerson, 1958; Kruskal & Wish, 1978; Shepard, 1980; Kriegeskorte & Mur, 2012). MDS plots of the behavioral RDMs illustrate perceived differences between videos based on different criteria (Fig. 1). For instance, in the transitivity RDM, categories such as “eating”, “tool use”, and “exercise” cluster together regardless of whether the depicted actions are social or nonsocial. By contrast, in the sociality-based behavioral RDM, social and nonsocial action videos are segregated into separate clusters, and distances among the social action videos are larger than differences among nonsocial videos. Related categories of social actions, such as “conversation” and people “eating” together are grouped into identifiable clusters in the sociality RDM but not in the transitivity RDM. To summarize, in total we tested nine representational models: motion, gaze, nonverbs, verbs, people, objects, scene, sociality, transitivity.

### Modeling neural representational geometry

**Figure 3.**
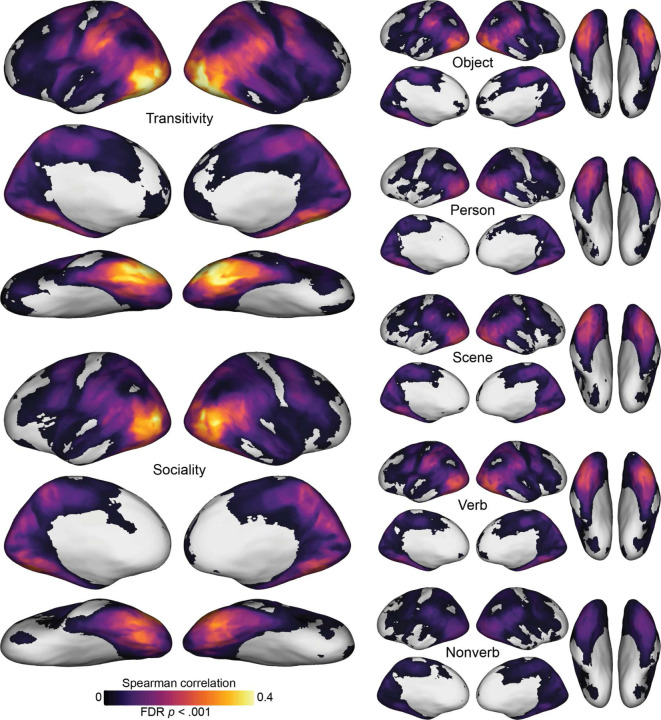
Searchlight correlation maps for behavioral and semantic models. Representational geometries for transitivity and sociality—based on behavioral judgments of the actions depicted in the video stimuli—were highly correlated with neural representational geometries throughout the action observation network, including lateral occipitotemporal, ventral temporal, inferior and intraparietal, and premotor cortices (left). Representational geometries for the object, person, and scene content in static images yielded qualitatively lower correlations (upper right), as did semantic geometries based on annotations with descriptive verbs and nonverbs (lower right). See Fig. S1 for gaze and motion-energy maps. Spearman correlation values were computed within each subject, averaged across subjects, and thresholded for statistical significance (permutation test, FDR controlled at .001).

We first separately computed the correlation between each model RDM and searchlight-based neural RDMs across cortex. Qualitatively, this analysis revealed that the representational geometries based on the purpose of depicted actions—the transitivity and sociality RDMs—are more strongly correlated with neural RDMs than are the other seven models (Fig. 3; Fig. S1). Significant correlations with these RDMs map out an extensive cortical system for the representation of agentic actions that includes most of the human visual system. This system includes lateral occipital cortex; temporal cortices in the ventral and inferior gyri and the superior temporal sulcus; parietal cortices in the inferior parietal lobe, intraparietal sulcus, and precuneus; and premotor cortices.

The transitivity RDM in particular, and to a lesser extent the object arrangement and verb RDMs, yields strong correlations extending into anterior parietal cortex (aIPS) and premotor areas (vPM). The sociality RDM yields a somewhat more focal map with highest correlations in posterior LO, extending into right pSTS, and bilateral VT. The transitivity RDM and sociality RDM both yield surprisingly strong correlations in VT, given this region’s typical association with face and object processing in experiments using static image stimuli. Transitivity outperforms sociality in frontoparietal areas and anterior VT, whereas sociality outperforms transitivity superior LO and pSTS, posterior VT, and precuneus/PMC (Fig. S2). Across essentially all of these maps, LO appears to have among the strongest correlations, corroborating its role as a hub of the action observation network that encodes a number of relevant features (Lingnau & Downing, 2015). The static-image arrangement RDMs (person, object, scene) and semantic RDMs (verb, nonverb) yield partially overlapping correlation maps, with generally lower correlation values. Of the image arrangement tasks based on visual contents, the numerically strongest correlations were observed for the object task, possibly due in part to the collinearity between the objects and the transitive nature of the actions. The analysis of correlations between neural RDMs and the word semantics of annotations showed that this approach produced much weaker correlations. Verb semantics, however, which generally describe the actions, were significantly stronger predictors of neural representational geometry than were nonverb semantics, which consist of nouns and adjectives that generally describe the objects, people, and scenes in the videos (Fig. S2).

**Figure 4.**
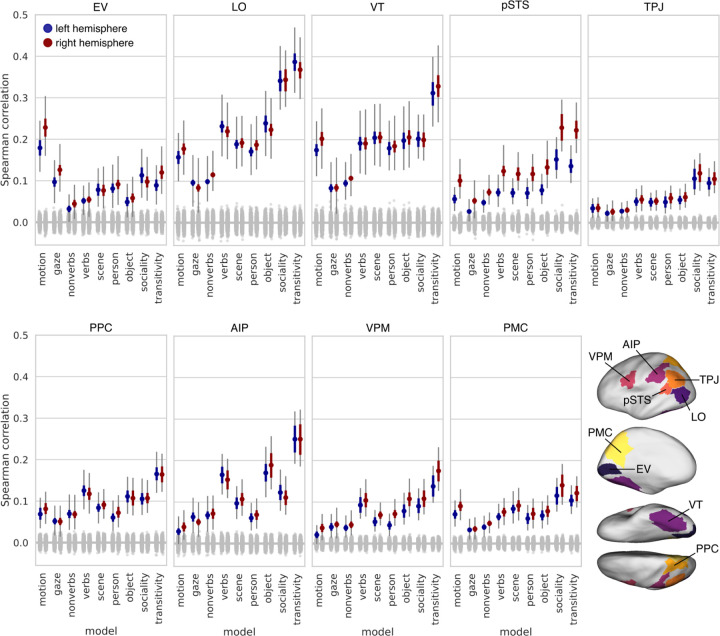
Model performance across nine regions of interest. ROIs were selected to span the visual processing hierarchy and encompass the action observation network. Colored dots indicate the mean Spearman correlation across subjects between a given model representational geometry and the neural representational geometry in that ROI. The gray null distribution is based on randomly permuting the 90 condition labels when constructing RDMs. Thick colored error bars indicate 95% bootstrap confidence intervals based on resampling subjects. Thin gray error bars indicate 95% bootstrap confidence intervals when resampling both subjects and stimuli. EV: early visual cortex; LO: lateral occipitotemporal cortex; VT: ventral temporal cortex; pSTS: posterior superior temporal sulcus; TPJ: temporoparietal junction; PPC: posterior parietal cortex; AIP: anterior intraparietal sulcus; VPM: ventral premotor cortex; PMC: posterior medial cortex.

We replicated the foregoing analysis using neural response patterns extracted from nine ROIs extending from early visual cortex into the action observation network (Fig. 4). As expected, motion energy was the strongest model in early visual cortex, exceeding all other models. Verb semantics and behavioral judgments emerged as the strongest models in LO and VT. Across all ROIs, behavioral judgments of transitivity and sociality were highest in LO. Transitivity was also the best-performing model in VT. Behavioral judgments of transitivity and sociality were also the best performing models in pSTS (particularly in the right hemisphere), TPJ, and PMC; sociality performed comparably to transitivity in LO, pSTS, TPJ, and PMC. Verb semantics performed well in dorsal areas like PPC, AIP, and VPM, and the object arrangement RDM performed well in AIP. That said, the transitivity model was the numerically strongest model across PPC, AIP, and VPM.

**Figure 5.**
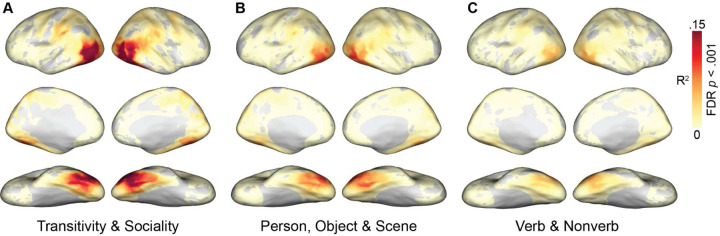
Joint model fit for different families of models. Multiple model RDMs were combined using multiple regression to quantify how much variance in neural representational geometry each family of models explains. Model fit was quantified as the proportion of variance explained (*R*^2^). (**A**) Model fit for a joint model comprising transitivity and sociality RDMs based on behavioral arrangement of dynamic video stimuli. (**B**) Model fit for a joint model comprising person, object, and scene RDMs based on behavioral arrangement of static images. (**C**) Model fit based on a joint model combining verb and nonverb semantic embeddings. *R*^2^ values were computed within each subject, averaged across subjects, and thresholded for statistical significance (bootstrap hypothesis test, FDR controlled at .001).

We next used a multiple regression analysis to combine multiple RDMs into three joint models: (1) a model combining the sociality and transitivity video-arrangement RDMs capturing the dynamic, action content of the clips; (2) a model combining the person, object, and scene image arrangement RDMs capturing the static, visual content of the clips; and (3) a semantic model combining verb and nonverb word embeddings. To evaluate these models, we computed the *R*^2^ for the joint model. Together, the behavioral RDMs for sociality and transitivity, accounted for a maximum of 22% of variance (*R*^2^ = .22) in searchlight neural representational geometries (Fig. 5). By contrast, the behavioral RDMs based on visual content—people, objects, and scenes—accounted for about half as much variance (maximum *R*^2^ = .12), and the semantic RDMs based on annotations accounted for about a third as much variance (maximum *R*^2^ = .07). In a direct comparison, the combined transitivity and sociality model outperformed the combined visual content model across essentially the entire action observation network, as well as in VT (Fig. 2).

To quantify the amount of reliable variance in neural RDMs (the noise ceiling) we calculated searchlight intersubject correlation (ISC) as the Spearman correlation between each participant’s neural RDM and the mean of other participants (Fig. S3). ISCs were strong in the same cortices that correlated with the action-purpose RDMs, with a maximum *r* = .75, indicating that the action-purpose RDMs accounted for almost 40% of the meaningful variance, as indexed by the noise ceiling, in neural representational geometry. The models capturing behavioral judgments of visual content (person, object, science) and the semantic annotation models peaked at 21% and 12% of the meaningful variance, respectively.

### Variance partitioning analysis

**Figure 6.**
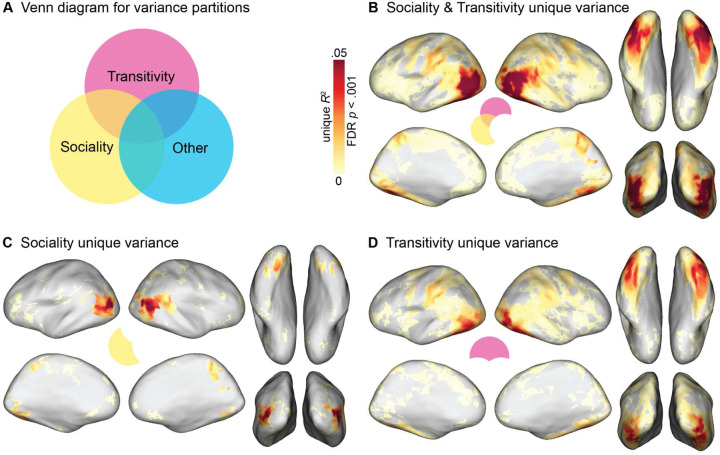
Unique variance explained by transitivity and sociality models. Hierarchical regression was used to quantify unique variance by models of interest: unique *R*^2^ = full *R*^2^ – nested *R*^2^, where the nested model excludes the model(s) of interest. The “other” partition comprises seven RDMs for person, object, and scene RDMs derived from static-image arrangements, verb and nonverb semantic RDMs, and gaze and motion-energy RDMs. (**A**) Venn diagram of schema for variance partitioning. (**B**) Unique variance explained jointly by sociality and transitivity RDMs. (**C**) Unique variance explained by sociality RDM. (**D**) Unique variance explained by transitivity RDM. Unique *R*^2^ values were computed within each subject, averaged across subjects, and thresholded for statistical significance (bootstrap hypothesis test, FDR controlled at .001).

To quantify how much variance in neural representational geometry can be uniquely explained by a given model RDM, we performed a variance partitioning analysis (Groen et al., 2018; Hebart et al., 2018). We used hierarchical regression to estimate the variance explained by a full model containing all models RDMs, and for nested models containing all models except a model (or models) of interest (Fig. 6A). We quantified the unique variance explained as: unique *R*^2^ = full *R*^2^ – nested *R*^2^, where the nested *R*^2^ excludes the model(s) of interest. First, we evaluated the unique variance explained by the RDMs derived from behavioral arrangements of dynamic video stimuli based on transitivity and sociality, accounting for all seven other models (person, object, scene image arrangements, verb and nonverb semantic embeddings, gaze trajectories, and motion energy). The combination of these two model RDMs uniquely explained variance throughout LO and VT (Fig. 6B), with a maximum unique *R*^2^ = .121 in LO. Remarkably, this exceeds the maximum *non*-unique variance explained by the static image arrangement RDMs (*R*^2^ = .118) and semantic RDMs (*R*^2^ = .068) reported in the previous section; that is, the video arrangement RDMs uniquely explain more variance (accounting for all other models) than other families of RDMs explain in total (including variance correlated with other models).

Finally, we separately evaluated the unique variance explained by the sociality RDM and the transitivity RDM. We found that the sociality RDM explained unique variance in posterior LO, right pSTS, posterior VT, and precuneus, with a maximum unique *R*^2^ = .073 in LO (Fig. 6C). The transitivity RDM explained unique variance across a more diffuse set of areas, including more anterior-inferior LO, AIP and vPM, as well as a more extensive expanse of VT, with a maximum unique *R*^2^ = .056 in lateral VT/LO (Fig. 6D). Overall, these findings indicate that both sociality and transitivity RDMs, based on the video arrangement tasks, capture a considerable amount of unique variance throughout cortex. The transitivity model captures a surprisingly large amount of unique variance in VT areas, including fusiform cortex, not typically associated with the perception of dynamic human actions.

## Discussion

Our findings indicate that behavioral judgments of the purpose and social content of actions capture neural representational geometries throughout the action observation network, including ventral temporal (VT) cortex. While the motion-energy model was the strongest model in early visual cortex (Fig. 4), behavioral judgments of transitivity and sociality emerged as the best-fitting models as early as LO, VT, and pSTS, and were superior models across much of cortex (Figs. 3–6). Behavioral judgments of the dynamic action-related content of the video stimuli (transitivity, sociality) outperformed behavioral judgments of the visual content of the stimuli (people, objects, scenes) conveyed by still images from the clips (Fig. S2). Our findings reveal surprisingly strong action representation in regions not typically associated with action understanding, particularly VT cortex, suggesting that the canonical action observation network (Decety et al., 1999; Grafton & Hamilton, 2007; Caspers et al., 2010; Rizzolatti & Sinigaglia, 2010; Oosterhof et al., 2013; Lingnau & Downing, 2015) should be expanded.

The transitivity model, reflecting behavioral assessments of object- and goal-directed action features, explained unique variance in neural representational geometry across a large swath of areas, including the frontoparietal network and a large portion of unique variance in inferior LO and anterior VT (Fig. 6). Transitivity was the numerically best-performing model in LO, VT, PPC, AIP, and VPM, highlighting privileged cortical representation for object- and tool-oriented actions (Martin et al., 1996; Chao et al., 1999; Beauchamp et al., 2002; Johnson-Frey, 2004; Peeters et al., 2009; Gallivan et al., 2013; Bracci & Peelen, 2013). The sociality model, on the other hand, captured neural representational geometry in a more localized set of areas, performing comparably to transitivity in several regions of interest (Fig. 4), and significantly exceeding transitivity in focal regions of superior LO, posterior VT, pSTS, and precuneus/PMC in the searchlight analysis (Fig. S2). The sociality model explained the largest portion of unique variance across both models in LO, as well as explaining unique variance in posterior VT, pSTS, and PMC (Fig. 6). We found that sociality captures unique variance in the superior-posterior extent of LO, whereas transitivity captures unique variance in the inferior-anterior extent of LO, corroborating prior work (Wurm et al., 2017). In a similar way, the sociality model uniquely explained variance in posterior VT, whereas transitivity uniquely explained variance in anterior VT. The verb model outperformed nonverbs in most areas, including LO and VT, and performed relatively well across frontoparietal areas (Kable et al., 2002, 2005; Bedny et al., 2008).

### Behavioral judgments of action meaning dominate cortical action representation

To obtain an assessment of the behaviorally-relevant features of actions based on human raters’ intuitions, we asked participants to arrange the video stimuli based on either the purpose (transitivity) or social content (sociality) of the depicted actions (C. E. Watson & Buxbaum, 2014; de la Rosa et al., 2014; Tucciarelli et al., 2019; Dima et al., 2022, 2023). We found that the combined performance of the transitivity and sociality models outstripped all other models, including low-level visual models, semantic models, and behavioral judgments of visual content (Fig. S2), capturing a large portion of unique variance throughout the action observation network, particularly in LO and VT (Fig. 5). This finding resonates with recent work by Contier and colleagues (2024) demonstrating that behavioral judgments of similarity (derived from a triplet odd-one-out task) among static images of objects provide a surprisingly strong model of functional tuning throughout visual cortex. Relatedly, Cichy and colleagues (2019) found, again using static object images, that behavioral judgments of perceived similarity capture rapidly emerging components of neural representational geometry that are not explained by categorical models or by bottom-up visual features learned by a deep convolutional neural network (DCNN). Taken together, these findings suggest that neural representations of both objects and actions, ranging from putatively low- to high-level cortical areas, are tightly yoked to the behavioral goals that guide judgments of similarity (D. D. Cox, 2014; Peelen & Downing, 2017; Bracci & Op de Beeck, 2023; Ritchie et al., 2024).

The previous argument, however, does not address our observation that behavioral judgments of action meaning (transitivity, sociality) in dynamic videos also better capture neural representational geometry than behavioral judgments of visual content (people, objects, scenes) in static images (Fig. S2). Part of this effect may be driven by the stimuli themselves: dynamic, naturalistic video stimuli more broadly and robustly engage the visual system than highly-controlled experimental videos or static images (Hasson et al., 2004; Fox et al., 2009; Haxby et al., 2020; Leopold & Park, 2020; Landsiedel et al., 2022; cf. Hafri et al., 2017). Certain kinds of meaning emerge from the dynamic evolution of visual features (e.g., Heider & Simmel, 1944) that are not explicit in static images. Behavioral judgments of dynamic clips may better align with neural activity encoding these dynamic features than behavioral judgments of corresponding still frames. That said, we suspect that part of the superiority of the transitivity and sociality models owes to the demands of the corresponding arrangement task. The transitivity and sociality arrangement tasks were designed to focus on two major dimensions of visual action understanding (Wurm et al., 2017; Dima et al., 2022) and orient participants toward the meaning or purpose of the actions depicted in the stimuli—that is, toward the “why” of action observation, that culminates in intentions (Blakemore & Decety, 2001; Van Overwalle & Baetens, 2009; Spunt et al., 2011) and social cognition more broadly (Frith & Frith, 2006; Adolphs, 2009; Quadflieg & Koldewyn, 2017). If the adaptive value of action perception is in ultimately understanding the physical and social ramifications of an observed action, then these intentional and social features of action may occupy a privileged role in cortical representation.

### Action representation in the ventral visual pathway

The prevailing model of the ventral visual pathway does not include representation of observed action (Goodale & Milner, 1992; Ungerleider & Haxby, 1994; Gauthier et al., 2000; Kanwisher, 2000; Haxby et al., 2001; Kriegeskorte, Mur, Ruff, et al., 2008; Kravitz et al., 2013; Grill-Spector & Weiner, 2014; Khaligh-Razavi & Kriegeskorte, 2014; Yamins et al., 2014; Güçlü & van Gerven, 2015; Bao et al., 2020). With this in mind, we expected the nonverb semantic model and the behavioral arrangement models based on person-, object-, and scene-related visual content to perform best in VT. Surprisingly, although these models were correlated with VT representation, they did not outperform more action-oriented models: the verb model yielded correlations roughly twice as large as the nonverb model; the sociality model performed on par with the person, object, and scene models; and the transitivity model outperformed all other models in VT by a large margin (Figs. 4, S2). Even when controlling for all other models, the sociality and transitivity models capture a large portion of unique variance in VT: the sociality model captures unique variance in posterior VT and relatively punctate regions of the fusiform gyrus; the transitivity model captures unique variance in a larger portion of VT extending more anteriorly (Figs. 6, S2).

These findings build on a line of work showing that VT encodes dynamic qualities of agentic behavior. For example, prior work has shown that lateral fusiform cortex responds to simple animations depicting agentic behavior (Castelli et al., 2000; Grossman & Blake, 2002; Gobbini et al., 2007) and goal-directed behaviors performed by automated manufacturing tools (Shultz & McCarthy, 2012). In recent work, Russ and colleagues have shown that homologous face-selective areas in macaque cortex are strongly driven by biological, socially-relevant motion (2015) and are highly sensitive to temporal continuity in dynamic, naturalistic videos (2022). Relatedly, in a study using naturalistic clips of behaving animals, Nastase and colleagues (2017) have shown that VT encodes action categories in a way that generalizes across the agents performing them. We contend that, taken together, these results demand a reassessment of VT function to incorporate observed action representation. We are not arguing that features like animacy (e.g., Sha et al., 2015), real-world size (e.g., Konkle & Oliva, 2012), and retinotopic bias (e.g., Silson et al., 2015), are not important dimensions of neural representational geometry in VT cortex—rather, we contend that VT also encodes dynamic features of agentic action, and that representations of all of these features are multiplexed during natural vision (Haxby et al., 2020).

### Action understanding for natural vision and behavior

The foregoing discussion raises some questions we believe are deserving of reflection. Why have we historically underestimated the role of behavioral judgments in shaping neural representational geometry? Why have we underestimated the importance of action representation in cortical areas like VT? With regard to the first question, one possible explanation is that we simply have not used a diverse enough, meaningful enough sample of stimuli to afford such rich behavioral structure (Dima et al., 2022, 2023; cf. Hebart et al., 2019, 2023, 2020; Contier et al., 2024 for encouraging alternatives in object representation). This limitation is likely a symptom of deeper biases in our approach, however. The vast majority of experiments in the action observation literature deliberately design stimuli and tasks so as to strip away as many confounding variables as possible; for example, brief videos of an isolated hand acting on a object in two different ways (e.g., Oosterhof et al., 2012; Wurm & Lingnau, 2015). The literature surrounding object representation in VT is by-and-large derived from contrasts between static images of isolated objects varying in particular ways (e.g., Kanwisher et al., 1997; Epstein & Kanwisher, 1998; Haxby et al., 2001). On the other hand, many experiments aiming to localize neural sensitivity to biological motion or agency deliberately strip away information about the form of agents and objects (e.g., Grossman et al., 2000; Gobbini et al., 2007; Walbrin et al., 2018).

Experiments following this “divide and conquer” strategy (Saxe et al., 2006) are useful in their own right, but they cannot give us a complete picture. We are trying to understand a system that is highly flexible and interconnected, that is exquisitely sensitive to context, and that is tuned to the statistical structure of the natural world (Hasson et al., 2020; Nastase et al., 2020). The “confounds” we strive to rule out in experimental manipulations are nearly ubiquitous in real-world contexts, and the brain appears to take advantage of these statistical dependencies (Simoncelli & Olshausen, 2001). This creates a dilemma: each individual experimental paradigm is designed to isolate and describe a particular piece of the puzzle—but this turns out to make the puzzle pieces very difficult to reassemble. For example, if we take an exquisitely face-selective puzzle piece (e.g., Tsao et al., 2006; L. Chang & Tsao, 2017) and try to fit it back into the “big picture” of dynamic, natural vision, this seemingly well-behaved puzzle piece suddenly changes shape (Russ & Leopold, 2015; Park et al., 2017; Russ et al., 2022). We encounter a similar dilemma in early visual cortex (David et al., 2004; Olshausen & Field, 2005). Experimenters design contrasts to minimize unwanted variance and manufacture a certain kind of data. Obviously this leaves little room for more nuanced similarities or differences between stimuli to emerge, but it also discards the statistical dependencies that may cut across puzzle pieces. When it comes to the brain, if we isolate a particular puzzle piece, it often changes shape. If we zoom in on a particular puzzle piece, it becomes difficult to judge its relative size; we run the risk of overestimating its importance relative to other puzzle pieces.

Naturalistic paradigms offer a way to reassemble these puzzle pieces: they force us to (re)examine each piece in its surrounding context, where the natural statistical dependencies cut across pieces, and allow us to better evaluate the relative contributions of different puzzle pieces to the larger picture (Haxby et al., 2020; Nastase et al., 2020). The current paradigm serves as a compromise between structured experiments and unconstrained videos or theatrical films (Huth et al., 2012; L. J. Chang et al., 2021; Richardson et al., 2018; Aliko et al., 2020; Lee Masson & Isik, 2021). Our use of arrangement tasks allows us to solicit relatively rich, continuous behavioral judgments without relying on predetermined annotations or questionnaires—for example, binary questions like “is this action directed at an object or set of objects?” (Tarhan & Konkle, 2020) or “is a social interaction present?” (Lee Masson & Isik, 2021), or Likert-scale ratings of questions like “are the people acting independently or jointly?” (McMahon et al., 2023). Our findings suggest that these behavioral arrangements better capture neural representational geometry than annotation-based semantic vectors (e.g., Huth et al., 2012).

The current work also has several limitations. First, our paradigm was not designed to examine individual differences in behavioral or neural representational geometry (Charest et al., 2014). Second, our passive viewing task with intermittent probe verbs during fMRI acquisition cannot speak to prior work showing that neural representational geometry flexibly reshapes to subserve particular task goals (Çukur et al., 2013; Bracci et al., 2017; Nastase et al., 2017; Shahdloo et al., 2022). Third, we do not test any artificial neural network models of visual processing among our selection of models (e.g., Lee Masson & Isik, 2021; McMahon et al., 2023). Our paradigm yielded a large amount of explainable variance in neural representational geometry, particularly in LO and VT, with a maximum of intersubject Spearman *r* = .75, (*R*^2^ ≈ .56) (compare to the similarly computed noise ceiling of Kendall’s τ = 0.26 for object images in VT reported by Khaligh-Razavi & Kriegeskorte, 2014). The combination of best performing models—transitivity and sociality—yielded a maximum *R*^2^ = .22 (*r* ≈ .47), corresponding to roughly 40% of the meaningful variance in neural representational geometry. This means that more than half of the meaningful variance in neural representational geometry remains unexplained. While we suspect neural network models may capture some portion of this variance, we are not overly optimistic as of yet. Jiahui, Feilong and colleagues (2023), for instance, have shown that state-of-the-art face recognition networks capture a relatively small fraction—3% at best—of the representational geometry in neural responses to dynamic, naturalistic videos of faces (and account for only 27% of variance in behavioral judgments of face similarity). Our findings suggest that the simple categorization or recognition tasks and static image stimuli used to train typical visual neural networks may not suffice to learn the dynamic, behaviorally-relevant features of human action representation. However, as neural networks advance to pursue more interactive, more social, more “human” objectives, they may very well close the gap.

## Methods

### Participants

Twenty-three right-handed adults (12 females; mean age ± SD: 27.3 ± 2.4 years) participated in the fMRI experiment. Each participant completed two 1-hour scanning sessions probing action representation, an additional 1-hour movie-viewing session outside the scanner (first half of the movie) immediately followed by a 1.5-hour movie-viewing session in the scanner (second half of the movie), and two 1-hour behavioral sessions. A subset of 17 of these participants then completed three follow-up behavioral sessions, each lasting approximately 1 hour. This amounts to roughly 3.5 hours of scanning time (including structural scans) and 8 hours of data collection in total per participant. A separate sample of 19 adults (11 females; mean age ± SD: 19.7 ± 2 years) participated in a 1-hour eye-tracking session. Two of these participants were excluded due to incomplete data collection. All participants gave written, informed consent prior to participating in the study. The study was approved by the Institutional Review Board of Dartmouth College.

### Stimuli and design

The stimuli for the main experiment consisted of 90 distinct video clips, each lasting 2.5 seconds, extracted from YouTube. These dynamic, naturalistic clips, featuring a wide variety of human behaviors within complex scenes, were selected to comprehensively sample neural representational space for observed action, span a broad spectrum of perceptual and semantic features, and elicit consistent responses across subjects (Bartels & Zeki, 2004; Hasson et al., 2004; Haxby et al., 2011; Huth et al., 2012; Sonkusare et al., 2019; Matusz et al., 2019; Nastase et al., 2020). The selected stimuli bridge the gap between the curated narratives of commercial cinema and the authenticity of real-world scenes.

The stimuli were categorized into 18 groups, delineated by social and nonsocial actions, with 5 exemplar clips per category. Social actions encompassed conversation, intimacy (e.g., hugging), teaching, and assembly-line work, while nonsocial actions included cooking, gardening, arts and crafts, and musical performance (e.g., playing an instrument). Additionally, five actions were classified under both social and nonsocial contexts: eating, dancing, exercising, cosmetics and grooming (e.g., hair styling, tooth brushing), and manual tool use (e.g., operating a power drill, using a saw). This categorization aimed to guarantee a diverse selection of content without participant awareness of the categorization or engagement in a categorization task. Despite the binary social/nonsocial categorization, some nonsocial clips contained social elements (e.g., bystanders). The selection deliberately varied in visual properties, the number of actors, and other semantic aspects such as actor gender and ethnicity, setting (indoor/outdoor), among others.

We developed a condition-rich, rapid event-related design, treating each of the 90 stimuli as a distinct experimental condition (Kriegeskorte et al., 2008). Each trial consisted of a 2.5-second video clip presentation followed by a jittered interstimulus interval featuring a fixation cross, averaging 2.5 seconds (Figure 1). Random variation in the jittered interstimulus fixation intervals was constrained such that no fixation interval was briefer than 2 s. The stimulus onset times were jittered using AFNI’s *make_random_timings.py* utility following an exponential decay curve (Ashby, 2011, pp. 84–86). For each participant, 1,000 random onset sequences were generated, evaluated for efficiency (Friston et al., 1999), and the most efficient design was selected.

To avoid overrepresented transitions between conditions by chance, type 1 index 1 serially balanced sequences were used to ensure that each trial type precedes and follows every other trial type (Aguirre, 2007). However, because a type 1 index 1 sequence is overly long for 90 conditions, we counterbalanced the presentation order of the 18 action categories. In addition to the 18 categories, we included null fixation trials and probe trials (described below) for 20 total trial types (amounting to ~5% fixation trials). A type 1 index 1 sequence for *n* = 20 trial types comprises *n*^2^ = 400 trials, with 360 trials of interest (excluding null fixation trials and probe trials). For a single participant, two unique type 1 index 1 trial orders were constructed and used for two scanning sessions on separate days (800 total trials, 720 trials of interest, 8 trials per stimulus, 4 trials per stimulus per session). For each trial order used, we first generated 1,000 sequences and selected the sequence with the highest efficiency. Trial sequences and timing were unique to each participant and session. Experimental stimuli were presented using PsychoPy (Peirce, 2007).

For a single scanning session, 400 trials were divided into four scanning runs of 100 trials each, totaling eight runs over two sessions. Runs were organized into blocks of 20 trials, where each of the 18 action categories, alongside a null fixation trial and a probe trial, was featured once in a randomized order. Each time an action category occurred, we randomly sampled without replacement from the five video clip exemplars for that category such that each exemplar occurred once per run. For each scanner run, the last three trials of the previous run (approximately 15 s of stimulation) were prepended to the beginning of the run to reinstate the temporal context of the sequence. The volumes acquired during these prepended trials were discarded prior to analysis. The first run of a session was prepended with the last three trials of the final run for that session. These three preparatory trials at the beginning of each run were sampled from a separate set of 18 clips (one for each category) not otherwise used in the stimulus set to ensure that no exemplar repetitions occurred in any run. An additional 5 s of fixation and 15 s of fixation were appended to the beginning and end of each run, respectively, making each run 535 s in duration, or approximately 9 minutes.

Participants were instructed to pay attention to the clips, and to keep their eyes on the fixation cross between clips. To ensure participants remained vigilant, we included probe trials using a two-alternative forced-choice semantic task. Prior to the experiment and before each run, participants were informed that they would occasionally be presented with two verbs and asked to answer the following question: “Which of these two verbs is most closely related to the action depicted in the preceding clip?” (see Table S1 for a full list of probe verbs). Probe trials occurred five times per run (once for each block of the type 1 index 1 sequence). The locations (i.e., left or right) of the particular verbs in a given pair were determined randomly per probe trial. This semantic probe was presented for 2.5 s and participants were instructed to respond during this period using either the left or right buttons of a single response box (held in the right hand). Participants were familiarized with the task and the format of the probe question prior to scanning.

The probe verbs were sampled from the WordNet lexical database (Fellbaum, 1990; Miller, 1995). For each implicit action category (as listed above, e.g., “conversation”), we retrieved four related verbs from WordNet (e.g., “argue,” “chat,” “converse,” and “discuss”). The related verbs were typically troponyms (i.e., subordinate verb categories) of some superordinate verb, e.g., “talk.” Troponyms were preferentially selected to minimize participant exposure to overarching categories. This means that most probe verbs were typically not a perfect description of the action depicted in the preceding clip, making the task non-trivial. The 80 probe verbs had a median depth of 3 (mean = 3.063, SD = 1.184) in the WordNet hierarchy, indicating that they were fairly specific subordinate categories. Note that verb hierarchies are generally shallower than noun hierarchies, rarely exceeding four levels (Fellbaum, 1990).

The structure of the type 1 index 1 sequences ensured that each action category was followed by a semantic probe probe trial once per scanning session. Because the five action categories depicted in social and nonsocial had related sets of verbs, we ensured that probe verbs from the social or nonsocial version of an action category were never paired (e.g., a verb from the social eating category was never paired with a verb from the nonsocial eating category). For trials where a semantic probe trial immediately followed a fixation trial or another probe trial (each necessarily occurring once per session due to the type 1 index 1 sequence), we replaced the trailing probe trial with a fixation trial. Behavioral responses (i.e., button presses) were monitored online during the scanning session to ensure task compliance. However, the log of recorded button presses was incomplete due to a technical error and therefore in-scanner behavioral responses were not further analyzed.

For the movie session, the film Raiders of the Lost Ark was split into 8 roughly 15 min segments. The segments were 840, 860, 860, 815, 850, 860, 860, and 850 s in duration. The first four movie segments were viewed outside the scanner immediately before the scanning session. Participants then freely viewed the latter four segments of the movie in the scanner.

### MRI acquisition

All functional and structural images were acquired using a 3 T Siemens Magnetom Prisma MRI scanner (Siemens, Erlangen, Germany) with a 32-channel phased-array head coil. Functional, blood-oxygenation-level-dependent (BOLD) images were acquired in an interleaved fashion using gradient-echo echo-planar imaging with pre-scan normalization, fat suppression, a multiband (i.e., simultaneous multi-slice; SMS) acceleration factor of 4 (using blipped CAIPIRINHA): TR/TE = 1000/33 ms, flip angle = 59°, bandwidth = 2368 Hz/Px, resolution = 2.5 mm3 isotropic voxels, matrix size = 96 × 96, FoV = 240 × 240 mm, 48 axial slices, anterior–posterior phase encoding. At the beginning of each run, three dummy scans were acquired to allow for signal stabilization. For each participant, eight runs were collected in two separate scanning sessions, each consisting of 535 volumes totaling 535 s (~9 min). At the beginning of each scanning session, a T1-weighted structural scan was acquired using a high-resolution single-shot MPRAGE sequence with an in-plane acceleration factor of 2 using GRAPPA: TR/TE/TI = 2300/2.32/933 ms, flip angle = 8°, resolution = 0.9375 × 0.9375 × 0.9 mm voxels, matrix size = 256 × 256, FoV = 240 × 240 × 172.8 mm, 192 sagittal slices.

For the movie session, both functional and structural images will be acquired using the parameters specified above. For each participant, four functional runs were collected. The eight runs are each roughly 15 min long, comprising 850, 860, 860, and 850 volumes (3,420 volumes in total).

### Preprocessing

All MRI data were preprocessed using fMRIPrep (Esteban et al., 2018). Anatomical images were skull-stripped using ANTs (Avants et al., 2008). Tissue-based segmentation isolating gray matter, white matter, and cerebrospinal fluid was implemented using FSL’s FAST (Zhang et al., 2001). For each participant, anatomical images were registered across sessions using FreeSurfer (Dale et al., 1999). Cortical surfaces were reconstructed from T1- and T2-weighted anatomical scans using FreeSurfer, spatially normalized based on sulcal curvature (Fischl et al., 1999). For the main experiment, a functional reference image was created for each participant using the median image after correction for head motion. Head motion parameters were estimated using FSL’s MCFLIRT (Jenkinson et al., 2002). Correction for slice-timing was performed using AFNI’s 3dTshift (R. W. Cox, 1996). Functional images were then aligned to the gray–white matter boundary of the T1-weighted anatomical image (estimated by FreeSurfer) in a single interpolation step using FreeSurfer’s 9-parameter affine boundary-based registration algorithm (Greve & Fischl, 2009). Functional data were projected onto the surface by averaging, at each surface vertex, values at six intervals sampled along a normal spanning the white matter and pial boundaries. Surface data were normalized to the fsaverage6 template with 40,962 vertices per hemisphere (81,924 vertices total).

### General linear model

Voxelwise general linear models were used to estimate response patterns for each of the 90 conditions. Stimulus-evoked response patterns for each event were modeled using a hemodynamic response function adjusted for a 2.5 s stimulus duration. The following nuisance variables were included in the model: six regressors accounting for head motion, 4th-order Legendre polynomial trends modeling slow baseline fluctuations, a regressor capturing framewise displacement (Power et al., 2012), and the first five principal components estimated from tissue-segmented cerebrospinal fluid (ventricle) time series (aCompCor; Behzadi et al., 2007). The first three preparatory trials, as well as the probe verb trials, were modeled with two additional nuisance regressors. Voxelwise models were estimated using AFNI’s 3dREMLfit, which accounts for temporal autocorrelation using an autoregressive–moving-average ARMA(1,1) model. Regression coefficients for each of the 90 conditions of interest were estimated across the four runs in each of the two scanning sessions; four trials contributed to each coefficient. This resulted in two sets of coefficients, one for each session. We z-scored response profiles across the 90 conditions for each voxel prior to further analysis.

For the movie session, voxelwise general linear models were used to regress out confounding variables. As above, nuisance variables included six regressors for head motion, a regressor for framewise displacement, 2nd-order Legendre polynomial trends, and the first five principal components from the ventricle time series. Bandpass filtering was also performed to remove temporal frequencies lower than 1/150 Hz and higher than 0.1 Hz. The regression model for the movie session was estimated using AFNI’s 3dTproject. The movie response time series at each voxel were z-scored prior to further analysis.

### Hyperalignment

We used hyperalignment to align individual-specific cortical-functional topographies into a common response space (Haxby et al., 2011). Specifically, we used a hybrid hyperalignment algorithm that estimates alignment parameters from a combination of both functional response time series and functional connectivity (Busch et al., 2021). We used a surface-based searchlight algorithm (20 mm searchlights) to construct a single whole-brain transformation (computed separately for each cerebral hemisphere) comprising locally-constrained transformation matrices (Guntupalli et al., 2016). Searchlight hyperalignment has previously been shown to improve the consistency of searchlight representational geometries across individual subjects (e.g., Nastase et al., 2017). Hybrid-hyperalignment parameters were estimated from data acquired in an independent movie scanning session where participants watched the second half of Raiders of the Lost Ark (~1 hour in duration and 3,420 volumes of movie-watching in total). These subject-specific transformations were then applied to the whole-brain response maps comprising the 90 coefficients estimated from the first-level regression model. All subsequent analyses were applied to response patterns hyperaligned into this common response space.

### Models of representational geometry

#### Motion energy.

To capture the dynamic, low-level visual features of each stimulus, we submitted each clip to a neurally-inspired motion-energy model (Adelson & Bergen, 1985; A. B. Watson & Ahumada, 1985; Nishimoto et al., 2011) implemented using *pymoten* (Nunez-Elizalde et al., 2021).

#### Gaze trajectories.

An independent sample of *N* = 17 subjects participated in the eye-tracking experiment, where they were presented with the 90 video clips from the main scanning experiment. Each clip was presented for 2.5 s while eye movements were recorded using an SR Research EyeLink 1000 Plus. Clips were presented in random order in four blocks with each stimulus occurring once per block (4 repetitions total). Participants were instructed to monitor for repetitions of the same clip stimulus, and 10 repetitions occurred per block. We directly analyzed the measured gaze trajectory (*x* and *y* coordinates over time). The trajectory was median filtered using a rolling window with a width of 84 ms and linear interpolation. Eye blinks indicated by the EyeLink software were censored and interpolated using the median filter (Wang et al., 2012). Trials where the eyes were closed for the entire duration of the stimulus were excluded, and blocks missing several or more trials (due to measurement error or participant compliance) were not further analyzed. Gaze trajectories were then decimated from 2500 samples at a sampling rate of 1000 Hz to 60 samples over 2.5 s (one sample per frame at a frame rate of 24 Hz).

For each block, the preprocessed gaze trajectories were used to construct an RDM capturing differences in spatiotemporal eye movement. To quantify the similarity of two gaze trajectories, we computed the Euclidean distance between the location of gaze (in two-dimensional screen coordinates) at each sample, and summed these distances across the stimulus presentation. To quantify the reliability of gaze trajectories, we first computed the Pearson correlation of gaze RDMs across blocks within each participant. In the sample of 17 participants, the mean inter-block pairwise correlation averaged across participants was .186 (SD = .137). To create a clean gaze RDM for further analysis, we excluded gaze RDMs from participants for which the inter-block (intra-participant) correlation of gaze RDMs was less than Pearson r = .1. Gaze trajectories for the remaining nine participants were used for all further analyses. The mean inter-block pairwise correlations for this subset of participants was .280 (averaged across participants, SD = .093). This suggests that there is modest consistency in each participant’s gaze allocation across blocks. To quantify inter-participant consistency of gaze trajectories, we averaged the gaze RDMs across blocks within each participant, then computed the Pearson correlation between these averaged gaze RDMs for each pair of participants. The mean inter-participant pairwise correlation of gaze RDMs was .390 (SD = .116 across pairs of participants), indicating that gaze RDMs are fairly consistent across individuals. The gaze RDMs for the remaining nine participants were averaged across blocks and across participants to construct a single gaze RDM. This average RDM was used in further analyses as a model of gaze allocation for the clip stimuli.

#### Word embeddings.

Two annotators manually assigned semantic labels to the 90 clip stimuli (as in Huth et al., 2012). Annotators were instructed to consider several factors when labeling the clips: person-related features such as gender, ethnicity, appearance, and body parts; object-related features such as tools used; scene-related features, such as indoor and outdoor contexts; and verbs describing the actions depicted (Kable et al., 2002; Bedny et al., 2008). Annotators were then instructed to select from this exhaustive set of labels the five most descriptive or salient labels for each clip. To quantify the semantic relationships among clips according to their assigned labels, we extracted 300-dimensional word embeddings from word2vec (Mikolov et al., 2013). We used pretrained semantic vectors based on the ~100 billion words Google News corpus. In this model, semantic relationships are encoded in the geometric relationships between word embeddings in a high dimensional vector space where more similar words are located nearer to each other.

To assess inter-annotator agreement, we assigned word embeddings to each of the top five labels selected by the two annotators and averaged these five vectors per stimulus, resulting in 90 semantic vectors for each of the two annotators. For each stimulus, we then computed the Pearson correlation between vectors for the two annotators. The average correlation between the two annotators across clips was .702 (SD = .123, range: .364–.942). This indicates substantial agreement between the two annotators. We then combined the two annotations and split the aggregated labels into verbs and nonverbs (person-, object-, and scene-related nouns and adjectives). The verb annotation comprised on average 3.311 words per stimulus (SD = 0.755, median = 3, range: 2–5 words), and the nonverb annotation comprised on average 4.722 words (SD = 0.633, median = 5, range: 3–6 words). For each stimulus, we separately averaged the verb embeddings and the nonverb embeddings. To construct verb and nonverb RDMs, we computed the cosine distance between the associated vectors for each pair of stimuli.

#### Behavioral arrangements.

From the original sample of fMRI participants (*N* = 23), 17 participants returned for five ~1-hour behavioral tasks after completing the scanning sessions. To acquire behavioral judgments of stimulus similarity, we used a multiple item arrangements paradigm (Goldstone, 1994; Kriegeskorte & Mur, 2012). Participants were presented with sets of stimuli positioned outside a large circle (or “arena”), and were instructed to organize these stimuli within the circle (see Fig. 2 for an example of the starting positions and a final arrangement).

Participants first performed two tasks in the two separate sessions: a “sociality” arrangement task and a “transitivity” arrangement task (Wurm et al., 2017), both related to the dynamic (inter)actions depicted in the video stimuli. The task/session order was counterbalanced across participants. In the sociality arrangement task, participants were instructed to organize the stimuli according to the social interaction depicted (if any). They received the following instructions: “Move the images into the circle and organize them so that clips depicting similar types of social interaction are nearest to each other. The more similar the two clips are in terms of social interaction, the closer the images should be.” In the transitivity arrangement task, participants were instructed to organize the stimuli according to the role of objects (if any) and the goal of the action. They received the following instructions: “Move the images into the circle and organize them so that clips in which similar objects play similar roles, and in which the actions have similar goals, are nearest to each other. The more similar the two clips are in terms of objects and goals, the closer the images should be.” A reminder of the task (“social interaction” or “object/goal”) was present in the upper left corner for the duration of the experiment. The task was self-paced and participants were verbally informed that they could ignore any task-irrelevant stimulus features.

Participants later performed three additional behavioral arrangement tasks in three separate sessions: a “person” arrangement task, an “object” arrangement task, and a “scene” arrangement task, all related to the visual features depicted in static-image frames from each original stimulus. The task/session order for the static-image arrangement tasks was also counterbalanced across participants. In the person task, participants received the following instructions: “Move the images into the circle and organize them so that images depicting similar people are nearest to each other. The more similar the people, the closer the images should be.” In the object task, they received the following instructions: “Move the images into the circle and organize them so that images depicting similar objects are nearest to each other. The more similar the objects, the closer the images should be.” In the scene task, they received following instructions: “Move the images into the circle and organize them so that images depicting similar scenes or places are nearest to each other. The more similar the scenery, the closer the images should be.”

For all arrangement tasks, participants were required to arrange 13 sets of stimuli. The first set included all 90 stimuli, while the subsequent 12 subsets each included 30 stimuli. We pseudo-randomly assigned stimuli to each of the 12 subsets (Goldstone, 1994). We generated random subsets by repeatedly permuting the list of stimuli and selecting subsets of 30 stimuli while recording the number of unique stimulus pairs occurring across each set of 12 subsets. We repeated this procedure 1,000 times and selected the set of 12 subsets with the greatest number of unique pairs. The stimuli were initially positioned at uniform intervals outside the arena, and the starting positions for each set of stimuli were determined randomly per participant. The final positions of the stimuli in each set were recorded after participants finalized their arrangement. Presenting all 90 stimuli in the first set served a dual purpose: first, participants were able re-familiarize themselves with the stimuli (both behavioral sessions occurred after the scanning sessions) and appreciate the scope of the stimulus set; second, this ensured that we acquired at least one measurement of the distance between every pair of stimuli. This first arrangement is inherently two-dimensional. However, aggregating distances from additional random subsets of the stimuli has been shown to afford psychological spaces exceeding two dimensions (Goldstone, 1994).

Each stimulus was represented by the first frame of the clip, cropped to a square and presented at 72 × 72 pixels. Stimulus images were resized so as to accommodate all 90 stimuli on the screen in the first set. Participants could use the mouse to either right-click and drag a stimulus to the desired location, or could left-click to select a stimulus and then click elsewhere to make the stimulus jump to the desired location. Finally, for the two video arrangement tasks (sociality and transitivity), participants could middle-click on a stimulus to inflate the stimulus to 256 × 144 pixels (the original aspect ratio) and play the 2.5 s video clip. In the static image arrangement tasks, the middle-clicked simply enlarged the still image. The experimental interface was created using PsychoPy (Peirce, 2007).

For each set of stimuli arranged by a participant, we computed the pairwise Euclidean distances between all stimuli in screen coordinates. For the first stimulus set containing all 90 stimuli, this yielded a full dissimilarity matrix. We then averaged the sparse pairwise distances computed from each subsequent random subset of stimuli with this dissimilarity matrix. Finally, we averaged the resulting RDMs across participants for subsequent analyses.

### Representational similarity analysis

We used representational similarity analysis (RSA) to evaluate different models of the neural representational spaces supporting naturalistic action understanding. As described in the previous section, we tested nine models of representational geometry: behavioral arrangements of dynamic videos based on (1) transitivity, (2) sociality, then semantics, behavioral arrangements of static images based on (3) person, (4) object, (5) scene contents, (6)verb, (7)nonverb, (8) gaze, and (9) motion energy. Each of these representational models is summarized into a 90 × 90 representational dissimilarity matrix (RDM) and embodies a hypothesized representational space.

We first used an exploratory surface-based searchlight analysis (10 mm radius) to map out the distribution of representational geometries throughout cortex (Kriegeskorte et al., 2006; Oosterhof et al., 2011). To construct neural RDMs, we computed the pairwise Pearson correlations between response patterns for each of the 90 action stimuli *across* the two scanning sessions (split-data RDMs; Henriksson et al., 2015; Walther et al., 2016), yielding a 90 × 90 neural RDM at each searchlight/cortical region. We first computed the intersubject correlation of searchlight representational geometries to demarcate an upper bound (or “noise ceiling”) of reliable variance in neural representational geometry (Nili et al., 2014; Nastase et al., 2019). For each searchlight, we computed the Spearman correlation between each participant’s RDM and the average RDM of the other participants and then averaged these correlation values (Fig. S1). To evaluate different representational models, for each searchlight/region, we computed the Spearman correlation between each participant’s neural RDM and each of the nine model RDMs. These correlation values were then submitted to a group-level statistical analysis. To quantify how well multiple RDMs jointly predict neural RDMs, we performed a multiple regression analysis: we ranked and standardized all model RDMs and used ordinary least-squares regression to predict the neural RDM. We quantified joint model performance using the coefficient of determination *R*^2^.

### Variance partitioning

To quantify the unique variance explained by a given model, accounting for variance explained by all other models, we performed a variance partitioning analysis. Using a hierarchical regression procedure, we first estimated a full model comprising all nine model RDMs; we then estimated a nested model containing all models except for the model(s) of interest. We quantified the unique variance explained by the model(s) of interest as the difference between the fit of the full model and the fit of the nested model excluding the model(s) of interest: unique *R*^2^ = full *R*^2^ – nested *R*^2^.

### Statistical evaluation

To evaluate the statistical significance of Spearman correlations between searchlight neural RDMs and model RDMs, we performed a permutation test: the signs of subject-level correlation values were randomly flipped at each permutation, Fisher-transformed, and averaged (10,000 permutations). Significance was assessed by quantifying the proportion of permuted statistical values that exceed the actual test statistic (Phipson & Smyth, 2010). For multiple regression analyses, the *R*^2^ values are positively biased above zero, resulting in overly permissive permutation distributions (or *t*-values). To more fairly assess the R2 values, we performed a bootstrap hypothesis test: we randomly sampled subject-level R2 values with replacement and recomputed the mean at each iteration (10,000 iterations), resulting in a bootstrap distribution around the test statistic; we then subtracted the actual test statistic from the bootstrap distribution, effectively re-centering it around zero in order to assess significance (Hall & Wilson, 1991). Searchlight statistical maps were corrected for multiple tests by controlling the false discovery rate (FDR) at .001 (Benjamini & Hochberg, 1995). For the ROI analysis, we computed null distributions by permuting the condition labels prior to constructing RDMs (i.e., shuffling the rows and columns of the RDM). We computed two kinds of bootstrap distributions: (1) we resampled subjects for typical population inference, and (2) we resampled both subjects and stimuli to assess generalizability across stimuli (Bedny et al., 2007; Nili et al., 2014; Westfall et al., 2016). All correlations were Fisher-transformed prior to averaging or statistical analysis, then converted back into correlations for visualization and numerical reporting.

## Supplementary Material

1
