# Supplementary material for: Behaviorally-relevant features of observed actions dominate cortical representational geometry in natural vision
Source: Res Sq. 2024 Dec 3:rs.3.rs-5478816. Preprint. [Version 1] doi: 10.21203/rs.3.rs-5478816/v1 (PMC11643331; doi:10.21203/rs.3.rs-5478816/v1)
Supplement: 1 [file NIHPPrs5478816V1-supplement-1.pdf]

Supplementary Information

Representational geometries supporting social action understanding in natural vision

Jane Han[1], Vassiki Chauhan, Rebecca Philip, Morgan Taylor, Heejung Jung, Yaroslav O. Halchenko, M. Ida Gobbini[2], James V. Haxby[1,*], Samuel A. Nastase[3,*]

[1] Department of Psychological and Brain Sciences, Dartmouth College, Hanover, NH, USA
[2] Department of Medical and Surgical Sciences (DIMEC), University of Bologna, Bologna, Italy
[3] Princeton Neuroscience Institute and Department of Psychology, Princeton University, Princeton, NJ, USA
[*] Equal contribution

Correspondence:
Jane Han: jane.han.gr@dartmouth.edu
James V. Haxby: james.v.haxby@dartmouth.edu
Samuel A. Nastase: snastase@princeton.edu

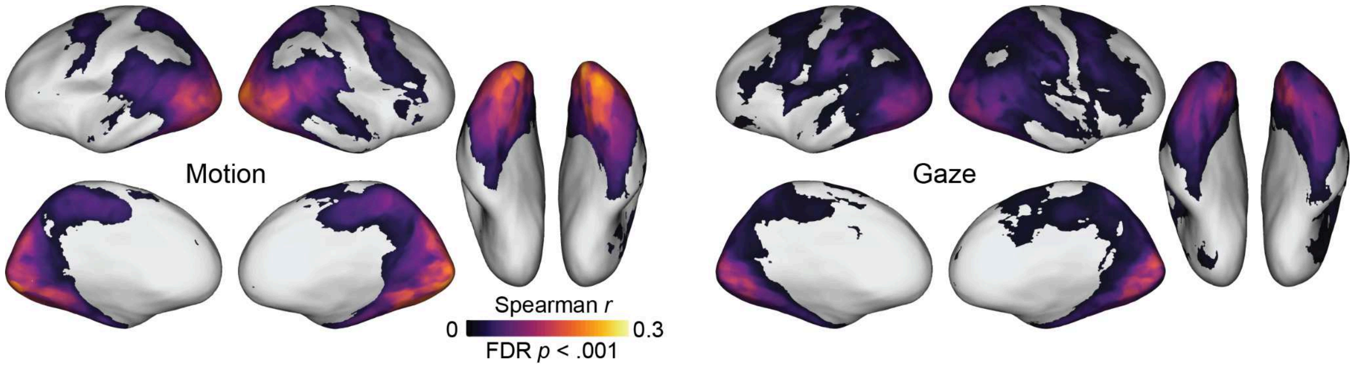

Figure S1. Searchlight correlation map for motion-energy and gaze models. The motion-energy RDM was derived from a biologically-inspired model of visual motion energy (Adelson & Bergen, 1985; A. B. Watson & Ahumada, 1985; Nishimoto et al., 2011). The gaze RDM comprised the Euclidean distances between gaze trajectories over the course of each stimulus clip measured using eye-tracking in a separate sample of subjects. Note that the maximum value of the color bar is set to Spearman $r = 0.3$ for visualization purposes, which is different from Fig. 3 where the maximum is 0.4. Spearman correlation values were computed within each subject, averaged across subjects, and thresholded for statistical significance (permutation test, FDR controlled at .001).

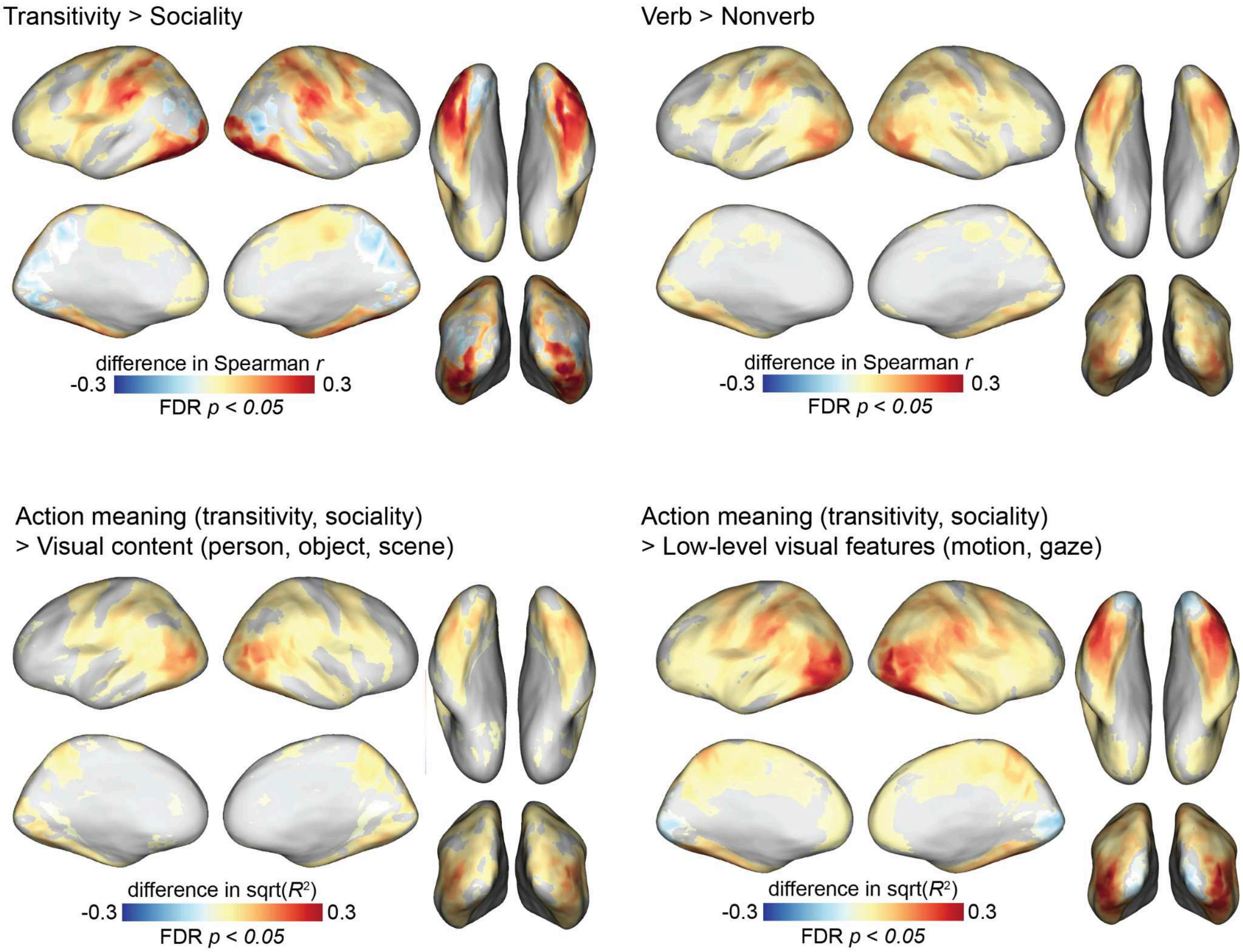

Figure S2. Comparing the performance of different model RDMs. To more explicitly compare models, we performed a paired *t*-test between model performance values (FDR controlled at .05). When comparing individual models (e.g., Transitivity > Socitality), we computed the mean difference between Spearman correlation values. When comparing joint models (e.g., Action meaning > Visual content), we computed the mean difference between R^2 values. To make the joint model comparison maps more comparable to the difference in correlations, we visualize the square-root of R^2 values.

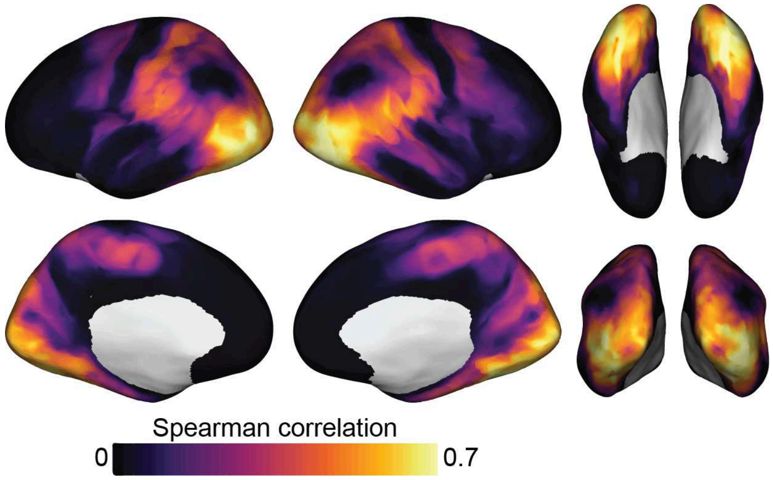

Figure S3. Intersubject correlation of searchlight representational geometries. To measure the reliability of neural representational geometries, we computed the intersubject Spearman correlation between each subject's searchlight RDMs and the average searchlight RDMs across the remaining subjects. This serves as an intersubject "noise ceiling" estimate of the maximum amount of meaningful variance available for modeling (Nili et al., 2014; Nastase et al., 2019). For visualization purposes, no statistical threshold or correction for multiple tests was applied.

Action category	Sociality	Probe verbs
Conversation	Social	argue, chat, converse, discuss
Intimacy	Social	caress, cuddle, embrace, hug
Teaching	Social	educate, instruct, lecture, teach
Manufacturing	Social	assemble, construct, labor, manufacture
Cooking	Nonsocial	bake, concoct, cook, roast
Gardening	Nonsocial	garden, harvest, plant, tend
Arts and crafts	Nonsocial	craft, design, fabricate, hand-build
Musical performance	Nonsocial	perform, recite, rehearse, serenade
Eating	Social	banquet, cater, dine, feast
Dancing	Social	choreograph, shimmy, synchronize, whirl
Exercise	Social	coach, invigorate, spot, train
Cosmetics and grooming	Social	beautify, clean up, make-over, spruce up
Tool use	Social	assist, cooperate, facilitate, operate
Eating	Nonsocial	devour, feed, nibble, swallow
Dancing	Nonsocial	gyrate, jive, pirouette, prance
Exercise	Nonsocial	exercise, exert, flex, revitalize
Cosmetics and grooming	Nonsocial	groom, preen, prim, primp
Tool use	Nonsocial	fix, handle, repair, wield

Table S1. Stimulus categories and probe verbs. We sampled 90 video stimuli depicting social and nonsocial actions in real-world contexts. Our goal was to sample the space of human actions as comprehensively as possible. The 90 individual stimuli were split into 18 different categories (left “Action category” column) with 5 exemplar clips per category. The 18 categories were split into social and nonsocial superordinate categories (middle “Sociality” column). Four categories were unique to the social category (Conversation, Intimacy, Teaching, Manufacturing) and four were unique to the nonsocial category (Cooking, Gardening, Arts and crafts, Musical performance). The remaining 10 categories comprised social and nonsocial versions of five action categories (Eating, Dancing, Exercise, Cosmetics and grooming, Tool use). Each category was associated with a set of four probe verbs (right “Probe verbs” column); during fMRI acquisition, participants were intermittently presented with two probe verbs (one matching the category of the previously-presented stimulus, and one randomly sampled from a different category) and asked to report which verb more closely corresponded to the action depicted in the preceding stimulus.
